# Dirty Money

**Published:** 1923-05

**Authors:** H. F. de La Flèchere


### Dirty Money.

Sir,—I have been greatly interested in the Editorial Note on this subject in your last issue. The dangers of handling dirty notes are obvious; but are those of handling coins much less serious? Less obvious they may be, but it cannot be doubted that many perils lurk in the dirt with which most coins are encrusted. The perilous trick of putting money into the mouth is enough to make a bacteriologist shiver—but then most things would! If anybody would seem more likely than another to suffer from the constant handling of money, it should surely be the bank clerk, yet, taken as a class, his health is, I believe, no worse than that of other people who are kept indoors. I observe, however, that when he is handling large numbers of notes, he takes the precaution of moistening his finger from a finger-bowl, instead of in the more primitive fashion.

H. F. de La Flèchere.

